# Hyperpolarized ^13^C urea myocardial first-pass perfusion imaging using velocity-selective excitation

**DOI:** 10.1186/s12968-017-0364-4

**Published:** 2017-06-21

**Authors:** Maximilian Fuetterer, Julia Busch, Sophie M. Peereboom, Constantin von Deuster, Lukas Wissmann, Miriam Lipiski, Thea Fleischmann, Nikola Cesarovic, Christian T. Stoeck, Sebastian Kozerke

**Affiliations:** 1 Institute for Biomedical Engineering, University and ETH Zurich, Gloriastrasse 35, 8092 Zurich, Switzerland; 2 Division of Surgical Research, University Hospital Zurich, Sternwartstrasse 14, 8091 Zurich, Switzerland

**Keywords:** Myocardial perfusion, First-pass perfusion, Hyperpolarization, 13C urea, Dynamic imaging

## Abstract

**Background:**

A velocity-selective binomial excitation scheme for myocardial first-pass perfusion measurements with hyperpolarized ^13^C substrates, which preserves bolus magnetization inside the blood pool, is presented. The proposed method is evaluated against gadolinium-enhanced ^1^H measurements in-vivo.

**Methods:**

The proposed excitation with an echo-planar imaging readout was implemented on a clinical CMR system. Dynamic myocardial stress perfusion images were acquired in six healthy pigs after bolus injection of hyperpolarized ^13^C urea with the velocity-selective vs. conventional excitation, as well as standard ^1^H gadolinium-enhanced images. Signal-to-noise, contrast-to-noise (CNR) and homogeneity of semi-quantitative perfusion measures were compared between methods based on first-pass signal-intensity time curves extracted from a mid-ventricular slice. Diagnostic feasibility is demonstrated in a case of septal infarction.

**Results:**

Velocity-selective excitation provides over three-fold reduction in blood pool signal with a two-fold increase in myocardial CNR. Extracted first-pass perfusion curves reveal a significantly reduced variability of semi-quantitative first-pass perfusion measures (12–20%) for velocity-selective excitation compared to conventional excitation (28–93%), comparable to that of reference ^1^H gadolinium data (9–15%). Overall image quality appears comparable between the velocity-selective hyperpolarized and gadolinium-enhanced imaging.

**Conclusion:**

The feasibility of hyperpolarized ^13^C first-pass perfusion CMR has been demonstrated in swine. Comparison with reference ^1^H gadolinium data revealed sufficient data quality and indicates the potential of hyperpolarized perfusion imaging for human applications.

**Electronic supplementary material:**

The online version of this article (doi:10.1186/s12968-017-0364-4) contains supplementary material, which is available to authorized users.

## Background

Cardiovascular Magnetic Resonance (CMR) perfusion imaging is widely used for the clinical assessment of myocardial ischemia and has proven its excellent diagnostic value [1]. To increase the sensitivity and specificity of detecting perfusion defects, imaging under pharmacologically induced stress is performed, followed by an optional measurement under rest condition [2]. In a clinical setting, stress is typically induced by administration of adenosine [3] or dobutamine [4].

Conventional first-pass perfusion CMR [5] is based on dynamic contrast enhancement (DCE) of saturation recovery [6] in response to bolus administration of a chelated gadolinium based contrast agent. Due to the exponential nature of spin-lattice relaxation and partial saturation effects of the image sequence itself, the relationship between signal and contrast agent concentration is non-linear, which renders the determination of actual contrast agent concentration non-trivial [7]. Using dual-bolus [8] or dual-sequences approaches [9] this issue can be addressed, but adds to complexity of data acquisition and analysis.

Despite a long history of safe use of gadolinium contrast agents in patients with normal kidney function, linear gadolinium based contrast agents have recently come under scrutiny in the wake of reports of gadolinium accumulation after repeated administration of certain types of agents [10–12]. To this end, alternative methods to assess tissue perfusion have attracted attention. Arterial spin labelling (ASL) has been applied to the heart in experimental and clinical settings [13]. Also, intra voxel incoherent motion (IVIM) measurements have allowed assessing perfusion fractions without the use of contrast agents [14–16]. However, both approaches provide limited contrast-to-noise ratios (CNR) compared to contrast-enhanced first-pass perfusion imaging and further research is required to translate these methods into clinical use.

Dissolution dynamic nuclear polarization (DNP) allows to produce solutions of highly polarized ^13^C–labeled molecules with >10′000-fold enhancement [17] relative to thermal signal. To compensate for the low abundance of carbon containing molecules in-vivo, a variety of endogenous ^13^C–labeled DNP substrates have been used for in-vivo imaging and spectroscopy studies, mostly probing metabolic pathways and their pathologic alterations in various organs and animal models [18]. Recently, first results of the application of hyperpolarized [1–^13^C] pyruvate to measure cardiac metabolism in humans have been reported [19].

Metabolically inert hyperpolarized substrates have been suggested as alternative contrast agents for perfusion measurements using DNP. HP001, a customized non-endogenous substrate has been shown to enable hyperpolarized perfusion assessment in rats [20]. Endogenous alternatives include the freely diffusible predeuterated 2-methylpropan-2ol (tert-butanol) [21], as well as α-trideuteromethyl [^15^N] glutamine with a particularly long T1 in-vivo [22]. Most prominently, ^13^C- labeled urea has been used to study perfusion in kidneys [23–26], liver [27, 28] and tumors [20, 28] in rodent and porcine models. In addition to qualitative assessments, the linear dependency of signal intensity on substrate concentration can simplify absolute blood flow quantification under certain assumptions [29]. Simultaneous probing of metabolism and perfusion with co-polarization of metabolically active and inert substrates may also improve diagnostic value by differentiation of metabolic and perfusion defects [30].

The feasibility of myocardial perfusion imaging using hyperpolarized ^13^C urea has recently been demonstrated in rodents [31]. The particular anatomy of the heart poses a major challenge: the close proximity of the myocardium to the left ventricular (LV) and right ventricular (RV) blood pools with comparatively high signal intensities causes signal contamination at practically achievable spatial resolutions. Accordingly, meaningful signal-intensity time curves are only obtainable if the blood pool signal is suppressed. To this end, flow-sensitizing gradients have been proposed to spoil signal contributions from the non-stationary blood pool [31]. This approach, however, depletes magnetization inside the blood pool, which in turn limits the achievable signal-to-noise ratio (SNR) inside the myocardium.

Dynamic metabolic imaging of hyperpolarized substrates in the heart typically relies on spectral-spatial excitation schemes, in which metabolites are excited depending on their chemical shift [32, 33]. Downstream metabolites are selectively excited with high flip angles, while the input substrate in the blood pool experiences reduced excitation angles. However, this approach is not applicable for metabolically inert substrates with a single resonance frequency, such as ^13^C urea.

In this work a velocity-selective tip-back excitation that mitigates polarization saturation inside the blood pool whilst retaining the suppression of blood pool signal is proposed. By integration of a bipolar velocity-encoding gradient into slice selection of a binomial excitation pulse, a velocity dependent phase shift in the blood pool signal is introduced, which can be utilized to preserve longitudinal magnetization in the blood pool. The method is validated using phantom measurements and the in-vivo applicability is demonstrated in swine. Myocardial signal-intensity time curves obtained with the proposed method are compared to conventional excitation and clinically used gadolinium enhanced perfusion CMR.

## Methods

### Sequence design

The accumulated phase *φ* of spins moving with velocity $\overrightarrow{v}$ subject to a bipolar gradient pair with amplitude $\overrightarrow{G}(t)$ is dependent on the first gradient moment ${\overrightarrow{m}}_1$:

1 $$ \varphi =\gamma\ \left({\overrightarrow{m}}_1 \cdot \overrightarrow{v}\right) $$

With

2 $$ {\overrightarrow{m}}_1=\int \overrightarrow{G}(t) t\; dt $$

As phase encoding only occurs along the gradient direction ${\widehat{e}}_G$, a scalar notation of velocities as projections of $v=\overrightarrow{v}\cdot {\widehat{e}}_G$ is used in this manuscript.

When combined with a binomial 1–1 excitation separated by the bipolar gradient pair, motion induced phase accrual can be exploited to tip back the magnetization of spins moving along the gradient direction by adjusting the excitation phase of the second radio-frequency (RF) pulse.

Alternatively, the accumulated phase between excitations for a given velocity *v*_*enc*_ can be set to *π* by scaling amplitude *G*(*t*) and duration *T* of the bipolar gradients such that:

3 $$ \gamma\ {v}_{enc}\ {\int}_0^T G(t) t\  dt=\pi $$

This approach does not require adjustments of the excitation phase for the second RF pulse with the additional benefit of being relatively robust towards small deviations from the nominal velocity *v*_*enc*_. Stationary spins inside the myocardium that do not accumulate motion-dependent phase effectively undergo two excitations, while the magnetization of moving spins inside the left ventricular blood pool is retained by tipping it back to the longitudinal axis by the second RF excitation. This excitation can be combined with any single-shot readout, such as echo-planar (EPI) or spiral imaging.

**Fig. 1:**
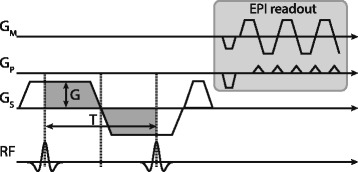
Velocity-selective excitation scheme: Bipolar velocity encoding gradients (*dark grey*) are incorporated into the slice selection gradients of a 1–1 binomial excitation. The accumulated phase of moving spins between the two RF pulses can be used to tip-back magnetization into the longitudinal axis with the second RF pulse. The approach is similar to spectrally selective binomial excitation, with the key difference that phase is induced by velocity encoding gradients instead of chemical shift variations of different molecules. The proposed excitation can be combined with arbitrary single-shot sampling approaches, such as echo-planar or spiral imaging

For through-plane motion, which is predominant for the left ventricular blood pool in short axis acquisitions, the bipolar gradients can be efficiently integrated into the slice selection scheme as illustrated in Fig. 1. Velocity components along other directions than through-plane will not be affected by the velocity-selective excitation and experience full excitation instead. In cases where the predominating direction of blood flow is not perpendicular to the slice orientation, the encoding gradients could be separated from the slice selection gradients and applied to different gradient axes at the cost of prolonged echo times.

**Fig. 2:**
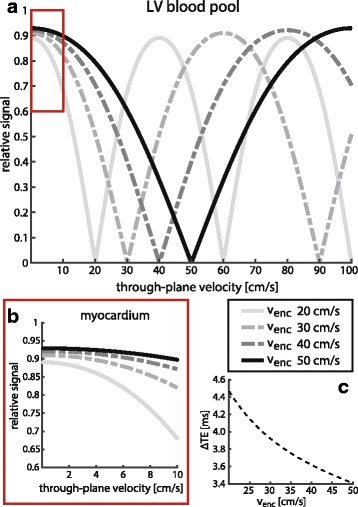
Calculated excitation patterns for velocity-selective excitation, relative to conventional single-pulse excitation. (**a**) effective excitation patterns for various encoding strengths inside the LV blood pool. Encoding of higher velocities yields a wider suppression band around the nominal values. (**b**) zoomed-in view for velocity range typically observed inside the myocardium (*red box, a*). For encoding strengths >30 cm/s the myocardial signal is hardly affected (<10%). (**c**) echo-time prolongation for velocity-selective excitation with respect to conventional single-pulse excitation for various encoding strengths

The effective excitation pattern for various *v*_*enc*_ encodings as a function of through-plane velocity over the imaging slice is illustrated in Fig. 2. For a given *v*_*enc*_ encoding, the relative signal amplitude *s* in dependency of the through-plane velocity *v* can be determined as:

4 $$ s(v)=\left| \sin \left(\frac{\left({v}_{enc}- v\right)}{v_{enc}}\frac{\pi}{2}\right)\right| $$

Conversely, the amount of preserved magnetization *M*_*z*_ in the blood pool can be estimated as:

5 $$ {M}_z(v)=\left| \cos \left(\frac{\left({v}_{enc}- v\right)}{v_{enc}}\frac{\pi}{2}\right)\right| $$

As the phase evolution is symmetric, the sign of the velocity and encoding gradients can be neglected. For the encoding velocity, the magnetization will be fully restored, while deviations due to spatial flow variations or mis-setting of the encoding velocity will result in imperfect tip-back and residual signal according to Eqs. 4 and 5 (see Fig. 2a). It is therefore crucial that encoding velocities are set to the specific mean systolic or diastolic velocity for each subject, which can be obtained from phase contrast pre-scans. Higher through-plane velocities in the LV blood pool, with higher respective *v*_*enc*_ encodings, benefit from a wider stop band around the target velocities (see Fig. 2a), whilst reducing unwanted signal dampening inside the slowly moving myocardium (see Fig. 2b). This makes the proposed excitation especially suited for stress perfusion measurements with increased blood velocities as presented in this work, but poses challenges for measurements under rest conditions. Additionally, echo time (TE) increases towards lower *v*_*enc*_ encodings, which limits the maximum attainable signal due to T_2_* decay (see Fig. 2c). The gap between the two RF pulses depends on encoding strength and gradient hardware and ranged from 6.8 ms (*v*_*enc*_: 50 cm/s) to 8.4 ms (*v*_*enc*_: 25 cm/s) on the clinical CMR system used in this study.

### CMR setup

All measurements were performed on a clinical 3 T Ingenia wide-bore scanner (Philips, Best, The Netherlands) equipped with a gradient system delivering 30 mT/m maximum amplitude at 200 T/m/s slew rate. Data on ^13^C were acquired using a custom-made 4-channel transmit/receive coil array (Clinical MR Solutions, Shadybrook, WI, USA) while the 2-channel body coil was utilized to sample on the ^1^H frequency. Animals were placed in right recumbency inside the scanner bore to allow for optimal placement of the coil arrays. A peripheral pulse oximeter unit was used for cardiac synchronization.

### Hyperpolarization

A 6.4 M ^13^C urea glycerol solution was prepared and doped with 18.5 mM AH111501 trityl radical. 1 mL samples were polarized for 4 h in a commercial 5 T SpinLab Polarizer (General Electric Healthcare, Waukesha, Wisconsin, USA) before dissolution using a buffer consisting of 30 mL 0.1% EDTA dissolved in water. Upon sample collection, the prepared solution was further diluted and cooled down with additional 4.5 mL of buffer at 0 °C to achieve body temperature for injection. 20 mL of the final 200 mM ^13^C urea solution were transferred to the scanner using an electromagnetic carrier device [34] built in-house, ensuring a magnetic field >7 mT over the whole syringe volume and transport period (15–20 s).

Polarization levels were established in separate experiments and amounted to 48 ± 5% at time of dissolution and 29 ± 3% at time of injection. T_1_ relaxation times inside the 3 T magnet were measured for the neat solutions (58 ± 1 s, *n* = 5) and after dilution in porcine heparinized venous blood samples within 15 min after sampling. Two urea concentrations (*n* = 5, each) were measured: 10% and 33%, with an observed T1 of 25 ± 2 s and 34 ± 0.5 s, respectively.

### Phantom validation

The proposed excitation scheme was validated using ^1^H imaging of a water tube embedded in a solid gel phantom. Water flow was adjusted to ~50 cm/s, which is in accordance with LV velocities typically observed in-vivo. A single 2D slice perpendicular to the direction of flow was acquired using a single-shot EPI readout. Signals inside the water tube as well as the stationary gel region were compared to simulations for the conventional single-pulse and the proposed tip-back excitation with varying *v*_*enc*_ encodings. All measurements were normalized to the water signal of the conventional excitation.

### Animal handling

Seven healthy female swine (Edelschwein, weight 30–35 kg) were used for the experiments. All swine were premedicated, intubated and sheaths (5 F) were introduced into both femoral arteries and veins. 100 IU/kgBW Heparin was given intravenously and repeated every hour. General anesthesia was maintained with isoflurane (2% - 3%) by positive pressure ventilation with 100% oxygen. Heart rate and rhythm and variability (ECG), inspiratory and expiratory gases (CO_2_, O_2_, isoflurane), pulse oximetry, temperature, direct arterial blood pressure, urine output, and arterial and venous blood gases were monitored continuously throughout the procedure. Hyperpolarized ^13^C urea and ^1^H gadolinium were administered through separate catheters in the femoral veins.

Cardiac stress was pharmacologically induced by intravenous administration of dobutamine (Dobutrex, TEVA Pharma AG, Basel, Switzerland) at increasing infusion rates until the target heart rate of 120 bpm was reached. Upon reaching the target heart rate, infusion rates were kept constant during all imaging experiments. 20 mL of hyperpolarized ^13^C urea solution was injected as a bolus at a rate of 10 mL/s. No adverse effects were observed as a result of ^13^C urea injections.

In one animal, acute myocardial infarction in the apical septum was induced by permanent distal ligation of the left anterior descending coronary artery (LAD). Corresponding data was not included in the main analysis of this study, but used as demonstration of sensitivity of the proposed excitation scheme to detect ischemic myocardium. After the procedure, all animals were euthanized in deep anesthesia by lethal injection of pentobarbital.

### In-vivo measurements

Prior to each ^13^C measurement, ^1^H phase-contrast images were acquired using a 2D short-axis cine sequence under stress condition. Sequence parameters were: FOV = 250 × 220 mm^2^, slice thickness 8 mm, in-plane resolution 2.5 × 2.5 mm^2^, TR = 4.8 ms, TE = 2.8 ms, FA = 7°, cardiac phases = 35, *v*_*enc*_ = 100 cm/s. These short-axis cine images were used to determine through-plane velocities and trigger delays for subsequent ^13^C perfusion CMR.

Anatomical reference 2D cine short-axis CMR was performed using a balanced steady-state free precession gradient echo sequence with the following parameters: FOV = 350 × 280 mm^2^, slice thickness 8 mm, in-plane resolution 1.9 × 1.9 mm^2^, TR = 2.7 ms, TE = 1.35 ms, FA = 45°, cardiac phases = 60.

Dynamic series of ^13^C perfusion images were acquired with cardiac triggering starting seven heart beats after the start of injection to minimize bolus saturation in the RV blood pool. Ventilation was suspended for the first 45 s of imaging to avoid misregistration between individual time frames. The proposed 1–1 binomial tip-back excitation (FA: 2 × 30°) was compared to single-pulse excitation (FA: 60°) in each animal under maintained stress condition with two hyperpolarized bolus injections, separated by 10 min. Identical single-shot echo planar imaging readouts were employed in both cases with following parameters: FOV = 110 × 110 mm^2^, slice thickness 15 mm, in-plane resolution 3.0 × 3.0 mm^2^, TR = 1 heartbeat, partial Fourier factor 0.65, readout duration 32 ms. Effective echo times for the proposed excitation increased from TE = 10.2 ms (conventional) to TE = 13.3–14.2 ms in dependence of flow encoding as illustrated in Fig. 2c. Encoding strengths (*v*_*enc*_) between 25 cm/s and 52 cm/s (mean ± std.: 38 ± 7 cm/s) for the velocity-selective excitation were used based on measured through-plane velocities.

Conventional ^1^H gadolinium contrast-enhanced perfusion CMR under stress was performed as a reference method in each animal. A dose of 0.1 mmol/kg gadolinium (Gadovist 1.0, Bayer, Zurich, Switzerland), followed by a 30 mL saline flush was injected at a rate of 4 mL/s using a power injector (Medrad, Indianola, PA, USA). Dynamic image series were acquired every heart beat using a saturation-recovery spoiled gradient echo sequence with parameters: FOV = 220 × 210 mm^2^, slice thickness 10 mm, in-plane resolution 3.0 × 3.0 mm^2^, TR = 1.9 ms, TE = 0.7 ms, FA = 15°, WET saturation [35] delay = 100 ms. A total of 100 dynamics were acquired during suspended ventilation.

### Image reconstruction

All in-vivo images were reconstructed from raw data using MRecon (GyroTools LLC, Zurich, Switzerland) and zero-filled to a common resolution of 1 × 1 mm^2^. ^1^H images were then rotated, aligned and cropped to the FOV of the ^13^C images. Nyquist-ghosts of the EPI readout were removed by first order phase correction maximizing signal intensity in a predefined region of interest (e.g. LV blood pool). ^13^C coil combination was implemented as root of weighted sum of squares [36].

### Post-processing

Left ventricular myocardium and LV blood pool were manually segmented on overlays of ^13^C perfusion and ^1^H reference images. The myocardium was subsequently divided into six segments corresponding to the basal/mid-ventricular slices in the 16 segment AHA model [37].

Coil sensitivities were estimated and corrected by fitting a plane over the myocardial signal at a time frame prior to and after bolus passage for ^1^H and ^13^C scans, respectively.

^13^C urea signal intensities were calculated as mean values over each segment and the LV blood pool. Global magnitude offsets were corrected for by subtraction of mean noise levels of the last 10 time frames in the image series.

Three-parameter gamma-variate functions

6 $$ s(t)= a\ {t}^b\ {e}^{-\frac{t}{c}} $$

were fitted to the signal time curves *s*(*t*) for further data analysis of ^13^C urea data. ^1^H gadolinium time curves were fitted with fourth order polynomials respectively. Area under the curve (AUC), peak myocardial contrast-to-noise ratio (pCNR) and up-slope were extracted from the fitted curves as semi-quantitative perfusion measures for ^13^C urea and ^1^H contrast-enhanced scans [7]. Signal-to-noise ratio (SNR) was calculated as mean signal intensities divided by the standard deviation over the difference of two noise frames pre (^1^H) or post (^13^C) bolus [38]. CNR was calculated as differences of peak contrast-enhanced SNR and mean baseline SNR as described in [39].

Absolute values and variability over the six myocardial segments were compared between conventional and the proposed tip-back excitation, as well as to ^1^H gadolinium based measurements. Analysis was restricted to first-pass signal contributions in all cases. Coefficients of variance (CoV) were calculated as standard deviation divided by mean values. A paired, two-sided Wilcoxon signed rank test was employed for statistical comparisons. A *p*-value <0.05 was considered significant.

## Results

### Phantom experiments

**Fig. 3:**
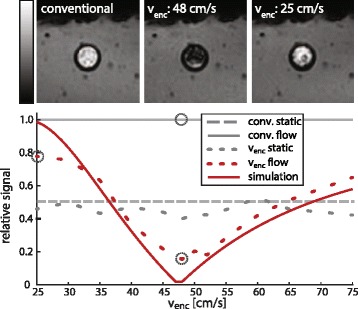
Phantom validation in ^1^H gel phantom around water pipe with mean flow velocity of 48 cm/s. (*top row*) Images acquired using a conventional single-pulse excitation (*left*), the proposed velocity-selective binomial excitation at nominal encoding strength (*center*) and half the nominal encoding velocity (*right*). (*bottom*) Measured vs. simulated signal intensities in static and flow compartments for varying velocity encodings. Data points corresponding to the *top images* are highlighted in *dashed circles*. Measured signal intensities overall match the predictions with maximum deviations of 20%. At nominal velocity encoding, residual signal stems from the low velocity regime at the boundary of the tube (see also Fig. 4). Encoding with half the nominal velocity restores most of the signal due to an accumulated phase of 2π. Deviations from nominal velocity and stronger T_2_* weighting for smaller v_enc_ values prohibit perfect recovery of magnetization in measurements

Figure 3 illustrates measured signal intensities of the proposed and conventional excitation in a phantom with constant flow of 48 cm/s in comparison to simulations. Variations in *v*_*enc*_ encoding strength show good agreement with the predicted signal overall. Encoding with the nominal velocity (48 cm/s) yields a > 80% reduction in water signal. Residual signal is observed at the tube boundary, where velocities deviate from the nominal value. Encoding with approximately half the nominal velocity (25 cm/s), which results in an accumulated phase of 2π, recovers 78% of the signal as expected due to inflow effects, stronger T_2_* weighting of the prolonged excitation and finite sharpness of the velocity distribution.

### In-vivo experiments

**Fig. 4:**
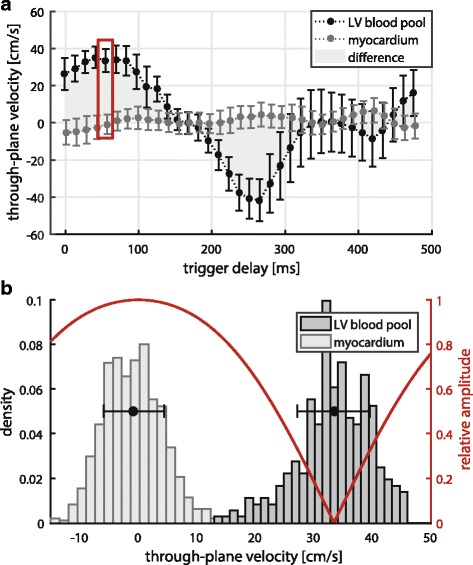
(**a**) measured through-plane velocities under dobutamine stress (120 bpm) over the cardiac cycle for *left* ventricular myocardium and blood pool. Mean and standard deviation over the respectively segmented areas are presented. Contrast of the proposed excitation is based on velocity differences between tissue and blood as highlighted in *grey*. Early systole (*red box*) and early diastole are therefore especially suited heart phases for the proposed method. (**b**) histogram of velocity distributions inside LV myocardium and blood pool in early systole (*red box, top plot*). Mean and standard deviation over both compartments are overlaid in *black*. Relative excitation amplitudes for encoding with the mean velocity inside the LV blood pool are shown in *red*. Blood pool signal is strongly suppressed whilst myocardial signal is largely retained

Figure 4a shows measured through-plane velocities over the cardiac cycle in short axis orientation under dobutamine stress. Variability of blood velocities between ejection phase (35 cm/s) and filling phase (− 42 cm/s) is observed in contrast to minor variations inside the myocardium (≤ 7 cm/s). The velocity difference between LV blood pool and myocardium is particularly pronounced in early systole and early diastole. Figure 4b illustrates the distributions of measured through-plane velocities of myocardium (−0.82 ± 5.2 cm/s) and LV blood pool (33.4 ± 6.2 cm/s) in early systole with the overlaid excitation pattern. Myocardial tissue gets excited with a relative amplitude >0.9, the LV blood pool with a relative amplitude <0.4 (within one standard deviation of the respective mean velocities). Preserved magnetization in the LV blood pool was estimated as 93 ± 4% over all subjects, based on spatial integration of Eq. 5. Saturation from sideband effects and blood exchange during excitation were considered negligible under in-vivo conditions.

**Fig. 5:**
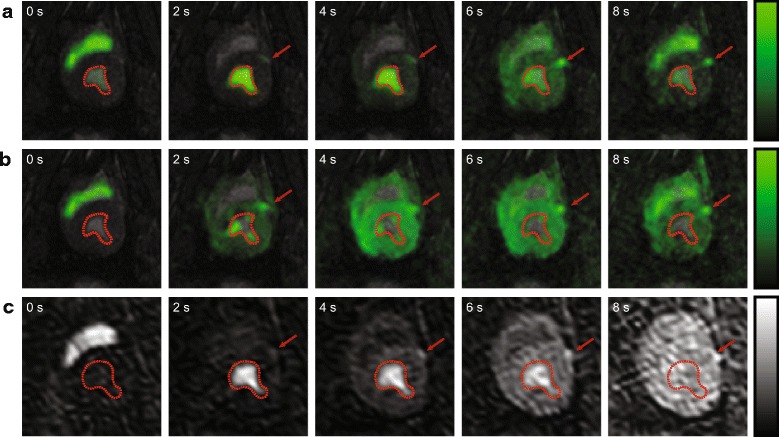
Dynamically enhanced cardiac perfusion imaging series covering bolus passage in LV blood pool and myocardium. (**a**) ^13^C urea conventional excitation. (**b**) ^13^C urea velocity-selective excitation. (**c**) ^1^H gadolinium based contrast agent. Image series were sequentially acquired in one animal under maintained stress conditioning. Myocardial bolus peak occurs between 4 and 6 s after injection (see Fig. 6). Left anterior descending coronary artery (LAD) is indicated by *red arrow*. LV blood pool is visualized by the *red contour*

In Fig. 5, a comparison of dynamic image series of myocardial bolus passage for ^13^C urea conventional excitation, ^13^C urea velocity-selective excitation and ^1^H gadolinium based contrast agent is shown. During LV bolus peak at 2 s (second column), blood pool signal suppression is apparent for the velocity-selective in contrast to conventional excitation. Residual LV signal stems from low blood flow near the trabeculae. Myocardial bolus signal (4–6 s) is significantly enhanced for the proposed method, with a clear distinction of right and left ventricular myocardium.

**Fig. 6:**
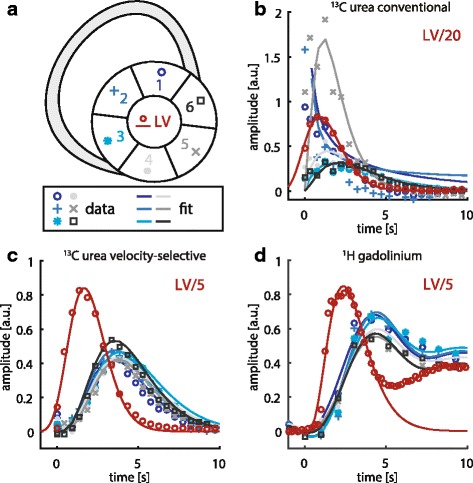
Representative comparison of perfusion curves extracted from a 6 sector AHA segmentation (**a**). Measurements were obtained within the same maintained dobutamine administration in the same animal for: (**b**) ^13^C urea conventional excitation. (**c**) ^13^C urea velocity selective excitation. (**d**) ^1^H gadolinium. Myocardial response data is presented as measured (*markers*) and fitted (*lines*). Arterial input data (*red*) is downscaled by factors 5 or 20 for better visualization. Gamma-variate fitting was employed for input curves and ^13^C urea response, 4th order polynomials were used for ^1^H gadolinium response

Figure 6 provides representative signal-time curves extracted from a six-sector AHA segmentation for ^1^H gadolinium (Gd), ^13^C velocity-selective (VS) and ^13^C conventional (C) measurements. Mean myocardial SNR averaged over each sector was 18.4 ± 1.5 (Gd), 25.7 ± 2.6 (VS) and 15.1 ± 2.5 (C), respectively.

For conventional excitation, blood pool spillover in the septal sectors 1 and 2, as well as the lateral sector 5 is observed. Myocardial peak signal appears at an unphysiologically early time point with respect to the LV peak bolus. Velocity-selective excitation yields uncontaminated myocardial response curves with good homogeneity over the six sectors. Myocardial peak signal is observed at the physiologically expected time point 2–3 s after the LV blood pool signal peak. Up-slope, timing and homogeneity are comparable with signal curves obtained from gadolinium-enhanced measurements.

**Fig. 7:**
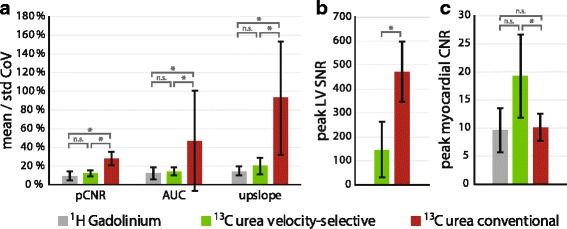
Analysis of semi-quantitative myocardial perfusion measures (pCNR, AUC, upslope) extracted from 6 sector AHA segmentation in six animals. ^1^H gadolinium, ^13^C urea velocity-selective excitation and ^13^C urea conventional excitation measurements were performed for each animal during maintained dobutamine administration. (**a**) Coefficient of Variance of semi-quantitative perfusion measures over the 6 myocardial AHA sectors (mean +/− std). (**b**) Absolute peak SNR of LV blood pool signal. (**c**) Absolute peak myocardial contrast-to-noise ratio for each modality (mean +/− std). n.s.: not significant, ^*^: *P* < 0.05

Intramyocardial coefficients of variance of semi-quantitative perfusion measures over six animals are evaluated for six animals in Fig. 7a. The variability of peak myocardial CNR is ≤20% for the three sequences (9 ± 5% (Gd), 12 ± 3% (VS), 20 ± 5% (C)). AUC variability is significantly (*P* = 0.03) higher for conventional excitation (47 ± 53%) compared to ^1^H gadolinium (12 ± 6%) and velocity-selective excitation (14 ± 4%). Upslope variability shows the largest inter-sequence discrepancy: 15 ± 5% (Gd), 20 ± 9% (VS), 93 ± 61% (C).

Peak SNR of LV blood pool is significantly (*P* = 0.03) lower for velocity-selective excitation (149 ± 117) compared to conventional excitation (473 ± 125), as illustrated in Fig. 7b.

Absolute myocardial peak CNR values are shown in Fig. 7c. The proposed excitation scheme shows a more than two-fold CNR increase compared to ^1^H gadolinium (19.2 ± 3.7 vs. 9.6 ± 3.9), and a significant (*P* = 0.03) increase compared to conventional excitation (9.5 ± 3.5).

### Acute infarction

**Fig. 8:**
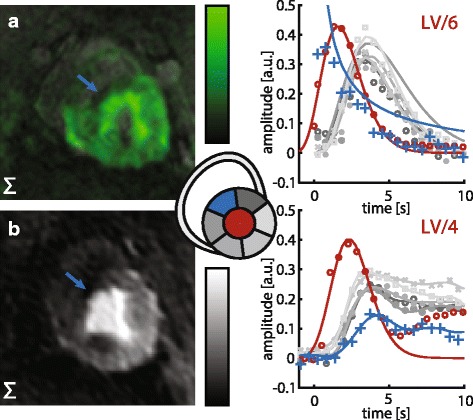
Stress perfusion measurements with septal infarction induced by permanent ligation of the distal anterior descending coronary artery. (*left*) Sum over three time frames at myocardial bolus peak. (*right*) corresponding perfusion curves for six AHA segments. Perfusion defect is visually observed in septal wall (*blue arrow*) and time curves of sector 2 (*blue curve*) for both modalities. LV signal spillover in the ^13^C urea perfusion curves stems from thinned myocardium in the infarct region. At myocardial bolus passage, the infarcted region shows strongly reduced signal. LAD is not detectable in either modality due to ligation. (**a**) ^13^C urea velocity-selective excitation. (**b**) ^1^H gadolinium based contrast agent

In Fig. 8 the feasibility of the proposed method to detect perfusion defects is demonstrated. In this case reduced septal perfusion is seen in both ^13^C velocity-selective and ^1^H gadolinium-enhanced images. Extracted myocardial perfusion curves confirm the perfusion defect in AHA sector 2 in both datasets. Increased LV signal spillover is detected for the urea measurement.

## Discussion

In this study, the feasibility of hyperpolarized myocardial first-pass perfusion CMR using velocity-selective excitation was demonstrated. Significantly enhanced SNR and CNR compared to conventional excitation (*P* < 0.05), as well as good discrimination of LV blood pool signal have been shown. Direct comparison with ^1^H gadolinium data indicates that the proposed method can produce sufficient data quality for diagnostic purposes in large subjects.

Comparison of velocity-selective ^13^C urea and ^1^H gadolinium measurements revealed good agreement between both methods. The signal enhancement during the myocardial first-pass showed similar patterns with respect to relative timing and signal amplitudes. Differences in signal-time curves were associated with differences in contrast agent characteristics. While gadolinium based agents showed a second bolus pass, the hyperpolarized signal vanished after the first-pass due to polarization decay and quenching by excitation. Accordingly, there was no baseline signal enhancement remaining at the end of the measurement, hence allowing individual hyperpolarized measurements to be performed in rapid succession, whereas gadolinium based contrast agents require a washout period. CNR and signal homogeneity of ^13^C urea imaging appeared to be sufficient for detecting perfusion defects as demonstrated in the ischemia example.

^1^H gadolinium measurements were performed using a relatively high contrast agent dose of 0.1 mmol/kg, which is at the upper end of clinical administration for qualitative and quantitative perfusion CMR. Despite this CNR-optimal dose, the average peak myocardial CNR was 50% lower than in velocity-selective ^13^C urea measurements.

Perfusion CMR in this study was limited to acquisitions under stress condition, while clinical protocols often include an optional reference measurement under rest condition. Precursor stages of coronary artery disease, however, are only detectable under increased myocardial workload [4], which gives stress perfusion a higher diagnostic value. Nonetheless, rest perfusion is required for the calculation of perfusion reserve as a semi-quantitative marker for myocardial ischemia that is widely used in clinical diagnostics [40]. Absolute quantification of myocardial blood flow (MBF) is a way of mitigating the need for additional measurements under rest conditions, whilst providing quantitative physiological parameters.

Due to limitations in scanner hardware, ^1^H gadolinium measurements had to be performed with the 2-channel body coil as a receiver, while a 4-channel surface coil was used for ^13^C urea detection. Consequently, image quality of ^1^H gadolinium data in this work was lower than in a standard perfusion CMR setup. A two-fold increase in SNR and 1.5-fold increase in CNR is estimated for measurements with a dedicated 28 channel cardiac coil array based on previous experiments. To this end, a dedicated ^1^H/^13^C receiver array will be commissioned to allow for improved ^1^H/^13^C signal detection in the future.

With the velocity-selective excitation scheme, the encoding gradients can in principle be scaled to any blood velocity. However, excitation profiles become narrower with lower velocities (see Fig. 2a), while measured velocity distributions remain relatively broad. This effect leads to a reduced effectiveness of the method under rest condition. Higher order velocity encoding could be used to broaden the suppression band of the excitation patterns in these cases. The efficacy of this approach given the necessary echo time prolongation needs to be investigated. During rest, the imaging window is meanwhile restricted to diastole, where rapid inflow of blood at >20 cm/s occurs. Preliminary acquired diastolic images appear sufficient for visual assessment of perfusion defects, however, the extraction of signal-intensity time curves is challenging due to the relatively thin myocardium (see Additional file 1: Figure S1).

Given a minimum TR < 50 ms, multiple slices within one cardiac cycle can be acquired. Depending on the number of slices and their distribution over the cardiac cycle, velocity encoding needs to be adjusted for each slice based on flow measurements at the respective heart phase (see Fig. 4b). While basal and mid-ventricular slices are well suited for velocity-selective excitation, acquisitions of apical slices remain challenging due to low through-plane velocities of blood in the apex. In order to utilize the radial blood flow pattern in the apical region, encoding along lateral directions might prove beneficial in this case. Encoding along directions orthogonal to the slice selection direction however results in additional echo time prolongation, as the encoding gradients must be separated from the slice selection gradients. Alternatively, encoding gradients may be adjusted to encode acceleration [41] in conjunction with a higher order binomial excitation. Despite the apparent echo time penalty, this approach could enable imaging in more heart phases and regions with non-uniform flow patterns such as the apex.

To maximize the efficacy of the proposed method, the bipolar gradient pair should be aligned with the predominant flow direction within the LV blood pool (basal to apical). Short axis acquisitions are therefore especially suited, as the encoding gradients can be partly integrated into slice selection gradients without echo time penalty. Nevertheless, the encoding gradients can be applied along directions other than the slice selection direction for applications where different slice orientations (e.g. long axis) are required. In addition to an echo time penalty from temporal gradient separation, this approach would also require an additional bipolar gradient along the slice selection direction to compensate for the phase accrual generated by the binomial excitation. The feasibility and efficacy of such an approach remain to be investigated.

Related to our work, the use of flow sensitizing gradients for the suppression of LV blood pool signal has previously been proposed [31]. In contrast to the signal dephasing of moving spins as in [31], the velocity-selective excitation proposed here has several advantages. Firstly, the magnetization inside the blood pool is preserved, which prevents pre-saturation of the hyperpolarized substrate bolus before its arrival in the myocardium. Consequently, large flip angles >45° can be applied to maximize signal in the first bolus passage. Secondly, dephasing the blood pool signal by means of bipolar gradients requires large first gradient moments, which also compromise myocardial signal.

As an alternative to the single-shot EPI readout used in this work, spiral k-space trajectories may be employed [30, 31, 42, 43]. However, the limited gradient performance of typical clinical systems renders sufficient in-plane resolution at reasonable field of views in large animals and humans challenging.

Qualitative and semi-quantitative perfusion CMR, as covered by this work, is diagnostically valuable for the detection of local ischemia. To reduce operator dependence during analysis and to enable the detection of absolute changes in perfusion, myocardial blood flow (MBF) quantification is preferred [7]. However, the determination of accurate arterial input functions from measured signals requires models of varying complexity [40, 44] and appropriate treatment of baseline signals. Hyperpolarized metabolically inactive substrates may present an advantage as the agent concentration is directly proportional to the measured signal upon correction for T_1_ decay and flip angle. Absolute perfusion quantification using hyperpolarized ^13^C substrates has been demonstrated in cancer, brain and kidneys [20, 28, 45]. These approaches are, however, not directly applicable to MBF quantification, as the myocardial response is affected by bolus saturation inside the blood pool. Future studies will combine velocity-selective imaging of the heart with interleaved sampling of the arterial input to address these issues.

## Conclusion

A velocity-selective excitation scheme for first-pass myocardial perfusion measurements using hyperpolarized ^13^C urea has been presented. Direct comparison with ^1^H gadolinium measurements showed very good agreement, indicating the potential of hyperpolarized ^13^C urea perfusion imaging for diagnostic purposes.

## Additional file

Additional file 1: Figure S1. Dynamically enhanced cardiac perfusion imaging series acquired under rest condition (heart rate = 90 bpm) in diastole using hyperpolarized ^13^C urea and velocity-selective excitation (*v*_*enc*_ = 25 cm/s). Myocardial bolus peak under rest condition is delayed by approximately 2 s compared with stress measurements due to lower heart rates. (PDF 517 kb)

## Abbreviations

AHA: American Heart Association (segmentation)
ASL: Arterial spin labelling
AUC: Area under the curve
CMR: Cardiovascular magnetic resonance
CNR: Contrast-to-noise ratio
CoV: Coefficient of variance
DCE: Dynamic contrast enhancement
DNP: Dissolution dynamic nuclear polarization
EPI: Echo planar imaging
FA: Flip angle
FOV: Field-of-view
IVIM: Intra voxel incoherent motion
LAD: Left anterior descending coronary artery
LV: Left ventricular
MBF: Myocardial blood flow
pCNR: Peak contrast-to-noise ratio
RF: Radio-frequency
RV: Right ventricular
SNR: Signal-to-noise ratio
TE: Echo time
TR: Repetition time
