# Supplementary figures and images for: Hyperpolarized 13C urea myocardial first-pass perfusion imaging using velocity-selective excitation
Source: J Cardiovasc Magn Reson. 2017 Jun 21;19:46. doi: 10.1186/s12968-017-0364-4 (PMC5480203; doi:10.1186/s12968-017-0364-4)

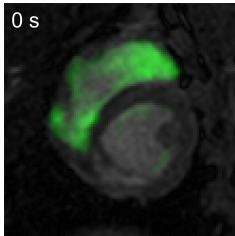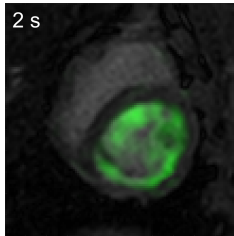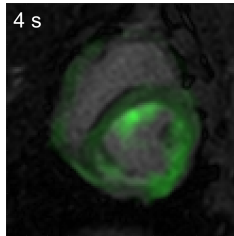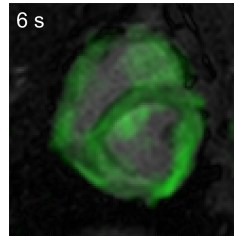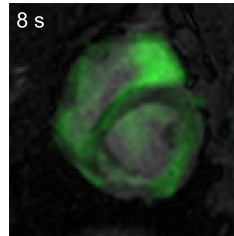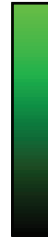

Supplement: Additional file 1: Figure S1. — Dynamically enhanced cardiac perfusion imaging series acquired under rest condition (heart rate = 90 bpm) in diastole using hyperpolarized 13C urea and velocity-selective excitation (v enc = 25 cm/s). Myocardial bolus peak under rest condition is delayed by approximately 2 s compared with stress measurements due to lower heart rates. (PDF 517 kb) [file 12968_2017_364_MOESM1_ESM.pdf]
